# Применение методов машинного обучения в дифференциальной диагностике АКТГ-зависимого эндогенного гиперкортицизма

**DOI:** 10.14341/probl13342

**Published:** 2024-02-27

**Authors:** О. О. Голоунина, Ж. Е. Белая, К. А. Воронов, А. Г. Солодовников, Л. Я. Рожинская, Г. А. Мельниченко, Н. Г. Мокрышева, И. И. Дедов

**Affiliations:** Национальный медицинский исследовательский центр эндокринологии; Национальный медицинский исследовательский центр эндокринологии; ООО «Статэндокс»; ООО «Статэндокс»; Уральский государственный медицинский университет; Национальный медицинский исследовательский центр эндокринологии; Национальный медицинский исследовательский центр эндокринологии; Национальный медицинский исследовательский центр эндокринологии; Национальный медицинский исследовательский центр эндокринологии

**Keywords:** гиперкортицизм, болезнь Иценко-Кушинга (БИК), АКТГ-эктопированный синдром, нейроэндокринная опухоль (НЭО), машинное обучение, прогностические модели

## Abstract

**ЦЕЛЬ:**

ЦЕЛЬ. Разработка неинвазивного способа дифференциальной диагностики АКТГ-зависимых форм эндогенного гиперкортицизма (ЭГ), а также оценка эффективности оптимального алгоритма прогнозирования вероятности АКТГ-эктопированного синдрома (АКТГ-ЭС), полученного с использованием методов машинного обучения на основе анализа клинических данных.

**МАТЕРИАЛЫ И МЕТОДЫ:**

МАТЕРИАЛЫ И МЕТОДЫ. В рамках одноцентрового, одномоментного, когортного исследования проведено ретроспективное прогнозирование вероятности АКТГ-ЭС среди пациентов с АКТГ-зависимым ЭГ. Пациенты случайным стратифицированным отбором разделены на 2 выборки: обучающую/тренировочную (80%) и тестовую (20%). Для построения прогностических моделей использовали 11 алгоритмов машинного обучения: линейный дискриминантный анализ (Linear Discriminant Analysis), логистическую регрессию (Logistic Regression), эластичную сеть (GLMNET), метод опорных векторов (SVM Radial), метод k-ближайших соседей (k-nearest neighbors, kNN), наивный байесовский классификатор (Naive Bayes), бинарное дерево решений (CART), алгоритмы дерева решений C5.0, бутстрэп-агрегирование CART (Bagged CART), случайный лес (Random Forest), градиентный бустинг (Stochastic Gradient Boosting, GBM).

**РЕЗУЛЬТАТЫ:**

РЕЗУЛЬТАТЫ. В исследование включены 223 пациента (163 женщины, 60 мужчин) с АКТГ-зависимым ЭГ, из них 175 пациентов с БИК, 48 — с АКТГ-ЭС. В результате предварительной обработки данных и отбора наиболее информативных признаков финальными переменными для классификации и прогнозирования АКТГ-ЭС отобраны: уровень АКТГ в 08:00, уровень калия (минимальное значение в активной стадии заболевания), показатели кортизола в суточной моче, кортизола в крови в 23:00, кортизола в слюне в 23:00, наибольший размер аденомы гипофиза по данным МРТ головного мозга. Наилучшую предсказательную способность из всех обученных моделей машинного обучения по всем трем итоговым метрикам — ROC-AUC (0,867), чувствительность (90%), специфичность (56,4%) — на тренировочной выборке продемонстрировала модель градиентного бустинга (Generalized Boosted Modeling, GBM). На тестовой выборке показатели AUC, чувствительности и специфичности модели в отношении прогнозирования АКТГ-ЭС составили 0,920; 77,8% и 97,1% соответственно.

**ВЫВОД:**

ВЫВОД. Прогностическая модель, основанная на методах машинного обучения, позволяет дифференцировать пациентов с АКТГ-ЭС и БИК по базовым клиническим результатам и может быть использована в качестве первичного скрининга пациентов с АКТГ-зависимым ЭГ.

## Abstract (en)

**AIM:**

AIM: To develop a noninvasive method of differential diagnosis of ACTH-dependent hypercortisolism, as well as to evaluate the effectiveness of an optimal algorithm for predicting the probability of ectopic ACTH syndrome (EAS) obtained using machine learning methods based on the analysis of clinical data.

**MATERIALS AND METHODS:**

MATERIALS AND METHODS: As part of a single-center, one-stage, cohort study, a retrospective prediction of the probability of EAS among patients with ACTH-dependent hypercortisolism was carried out. Patients were randomly stratified into 2 samples: training (80%) and test (20%). Eleven machine learning algorithms were used to develop predictive models: Linear Discriminant Analysis, Logistic Regression, elastic network (GLMNET), Support Vector machine (SVM Radial), k-nearest neighbors (kNN), Naive Bayes, binary decision tree (CART), C5.0 decision tree algorithms, Bagged CART, Random Forest, Gradient Boosting (Stochastic Gradient Boosting, GBM).

**RESULTS:**

RESULTS: The study included 223 patients (163 women, 60 men) with ACTH-dependent hypercortisolism, of which 175 patients with Cushing’s disease (CD), 48 — with EAS. As a result of preliminary data processing and selection of the most informative signs, the final variables for the classification and prediction of EAS were selected: ACTH level at 08:00 hours, potassium level (the minimum value of potassium in the active stage of the disease), 24-h urinary free cortisol, late-night serum cortisol, late-night salivary cortisol, the largest size of pituitary adenoma according to MRI of the brain. The best predictive ability in a training sample of all trained machine learning models for all three final metrics (ROC-AUC (0.867), sensitivity (90%), specificity (56.4%)) demonstrated a model of gradient boosting (Generalized Boosted Modeling, GBM). In the test sample, the AUC, sensitivity and specificity of the model in predicting EAS were 0.920; 77.8% and 97.1%, respectively.

**CONCLUSION:**

CONCLUSION: The prognostic model based on machine learning methods makes it possible to differentiate patients with EAS and CD based on basic clinical results and can be used as a primary screening of patients with ACTH-dependent hypercortisolism.

## ОБОСНОВАНИЕ

Эндогенный гиперкортицизм — крайне редкое и одно из наиболее тяжелых эндокринных заболеваний, обусловленное длительным воздействием на организм избыточного содержания кортизола. Во избежание развития жизнеугрожающих осложнений и инвалидизации больных необходима своевременная диагностика данного состояния с установлением этиологии и последующим проведением адекватного лечения [1]. Среди спектра нозологий, приводящих к гиперкортицизму, до 80% случаев составляют АКТГ-секретирующие аденомы гипофиза (болезнь Иценко-Кушинга (БИК)). Значительно более редким вариантом АКТГ-зависимого гиперкортицизма является АКТГ-эктопированный синдром (АКТГ-ЭС), обусловленный избыточной продукцией АКТГ, реже кортикотропин-рилизинг гормона (КРГ), нейроэндокринной опухолью (НЭО) различной локализации [2].

Дифференциальная диагностика БИК и АКТГ-ЭС представляет наибольшую сложность, особенно у пациентов без визуализации аденомы или с микроаденомами гипофиза менее 6 мм в диаметре и/или отрицательными результатами топической диагностики НЭО, по данным методов лучевой диагностики, функциональной и рецепторной визуализации [3].

«Золотым стандартом» дифференциальной диагностики АКТГ-зависимых форм эндогенного гиперкортицизма является двусторонний селективный забор крови из нижних каменистых синусов с определением градиента АКТГ центр/периферия до и после введения стимуляционного агента [4]. С момента внедрения метод претерпел значительные изменения и усовершенствования с целью повышения диагностической точности [5]. Результаты исследования, проведенного в ФГБУ «НМИЦ эндокринологии» Минздрава России, свидетельствуют о высокой чувствительности (98,31% (95% ДИ 95,16–99,43)) и специфичности (94,64% (95% ДИ 85,39–98,16)) селективного забора крови с использованием стимуляции десмопрессином [6].

Несмотря на высокую диагностическую точность метода, селективный забор крови из нижних каменистых синусов осуществляется в единичных медицинских учреждениях Российской Федерации (РФ) ввиду технических сложностей данной процедуры, высокой стоимости, трудозатратности и потенциальной возможности осложнений инвазивного вмешательства. В свою очередь используемые для дифференциальной диагностики неинвазивные диагностические тесты, такие как большая дексаметазоновая проба (БДП), периферическая проба с кортиколиберином, обладают недостаточной чувствительностью и специфичностью [7]. Для улучшения дифференциальной диагностики АКТГ-зависимых форм эндогенного гиперкортицизма очевидна необходимость поиска более доступных, удобных в применении и неинвазивных методов, которые могут быть использованы в условиях невозможности проведения селективного забора крови из нижних каменистых синусов.

В связи с развитием цифровых технологий и курсом на персонализированную медицину в последние годы наблюдается тенденция к активному внедрению методов искусственного интеллекта для реализации поддержки принятия врачебных решений во многих областях медицины [8]. Методы машинного обучения относят к основным инструментам искусственного интеллекта и все чаще используют в диагностических и прогностических исследованиях. Для решения проблемы ранней диагностики и дифференциальной диагностики АКТГ-зависимого гиперкортицизма разработка алгоритма, сочетающего анализ простых анамнестических данных, результаты клинического и инструментального обследования при отсутствии инвазивности может быть предложен в качестве метода скрининга или первичной диагностики.

## ЦЕЛЬ ИССЛЕДОВАНИЯ

Разработка неинвазивного способа дифференциальной диагностики АКТГ-зависимых форм эндогенного гиперкортицизма на основе параметров, доступных в обычной клинической практике, и оценка эффективности оптимального алгоритма прогнозирования вероятности АКТГ-ЭС, полученного с использованием методов машинного обучения.

## МАТЕРИАЛЫ И МЕТОДЫ

## Дизайн исследования

Одноцентровое, одномоментное, когортное исследование с ретроспективным анализом данных. В исследование включены пациенты, находившиеся на стационарном обследовании и лечении в отделении нейроэндокринологии и остеопатий ФГБУ «НМИЦ эндокринологии» Минздрава России в период с 28.10.2015 по 29.12.2022 гг.

## Критерии соответствия

Критерии включения в исследование: пациенты обоих полов с АКТГ-зависимым эндогенным гиперкортицизмом, подтвержденным в соответствии с федеральными клиническими рекомендациями [7] как минимум двумя лабораторными тестами: повышение свободного кортизола в суточной моче и/или кортизола в образце слюны, собранной в 23:00, или кортизола крови в 23:00, и/или отрицательная малая проба с 1 мг дексаметазона (отрезная точка 50 нмоль/л); концентрация АКТГ в утренние часы ≥10 пг/мл; отсутствие визуализации аденомы гипофиза на МРТ или размеры аденомы гипофиза менее 10 мм.

Критерии исключения из исследования: пациенты с другими верифицированными формами гиперкортицизма; двусторонняя адреналэктомия в анамнезе; прием препаратов для консервативного лечения гиперкортицизма; терапия аналогами соматостатина пролонгированного действия; женщины в период беременности, родов, грудного вскармливания; лица, страдающие психическими заболеваниями; пациенты с неудовлетворительно заполненной медицинской документацией; пациенты с отсутствием информации по анализируемым показателям (пропущенными значениями).

## Способ формирования выборки из изучаемой популяции

Для построения модели дифференциальной диагностики БИК и АКТГ-ЭС использовали данные выборки пациентов с АКТГ-зависимым эндогенным гиперкортицизмом, сформированной из общей когорты пациентов, прошедших стационарное обследование и нейрохирургическое лечение в ФГБУ «НМИЦ эндокринологии» Минздрава России или хирургическое лечение НЭО с эктопической продукцией АКТГ в специализированном медицинском учреждении соответствующего профиля в 2015–2022 гг.

Для формирования выборки применяли следующие критерии включения пациентов в выборку: пациенты с проведенным нейрохирургическим лечением по поводу БИК, достигшие ремиссии заболевания и/или наличие гистологического и иммуногистохимического подтверждения диагноза; пациенты с проведенным хирургическим лечением по поводу НЭО с эктопической продукцией АКТГ, достигшие ремиссии заболевания и/или наличие гистологического и иммуногистохимического подтверждения диагноза; пациенты без проведенного хирургического лечения, с отсутствием градиента АКТГ центр/периферия по результатам селективного забора крови из нижних каменистых синусов, с наличием признаков НЭО по данным планарной сцинтиграфии в режиме сканирования «всего тела», совмещенной с ОФЭКТ/КТ, с 99mTc-тектротидом и/или совмещенной позитронно-эмиссионной и компьютерной томографии с DOTA-конъюгированными радиофармпрепаратами (68Ga-DOTA-TATE, 68Ga-DOTA-TOC, 68Ga-DOTA-NOC).

Критерии исключения пациентов из выборки: пациенты без проведенного нейрохирургического или хирургического лечения и с отсутствием гистологической верификации диагноза или признаков НЭО по данным методов лучевой и радионуклидной диагностики.

## Описание медицинского вмешательства

У всех больных, включенных в исследование, на этапе скрининга лабораторно и клинически подтвержден АКТГ-зависимый эндогенный гиперкортицизм в соответствии с клиническими рекомендациями [7]. С целью дифференциальной диагностики БИК и АКТГ-ЭС 113 пациентам, включенным в исследование, проводилась БДП. Снижение кортизола крови в 08:00 более чем на 60% от исходного после приема накануне 8 мг дексаметазона расценивалось как центральная форма эндогенного гиперкортицизма (БИК), тогда как снижение уровня кортизола менее чем на 60% свидетельствовало в пользу АКТГ-ЭС.

Всем пациентам, включенным в исследование, выполнялся селективный забор крови из нижних каменистых синусов на фоне стимуляции десмопрессином (Desmopressin Acetate 4 мкг в 1 мл для в/в, в/м и п/к инъекций Ferring Pharmaceuticals) в дозе 8 мкг. Результаты селективного забора крови оценивались на основании расчетного максимального отношения уровней АКТГ в синусах к периферическому уровню АКТГ до стимуляции десмопрессином и после введения препарата внутривенно. Градиент АКТГ центр/периферия ≥2 до стимуляции и/или ≥3 после стимуляции десмопрессином свидетельствовал в пользу БИК, более низкие значения градиента АКТГ центр/периферия расценивались как АКТГ-ЭС [9].

## Методы регистрации исходов

МРТ головного мозга проводилась на магнитно-резонансном томографе Magnetom Harmony (Siemens, Германия) с напряженностью поля от 1,5 до 3 Тесла с введением гадолиниевого контрастного препарата.

Гормональное исследование АКТГ (референсный интервал: утро 7,2–63,3 пг/мл, вечер 2–25,5 пг/мл), кортизола в сыворотке крови в 23:00 (64–327 нмоль/л), определение свободного кортизола в вечерней слюне (0,5–9,6 нмоль/л) проводилось электрохемилюминисцентным методом на анализаторе Cobas 6000 Module e601 (Roche); измерение свободного кортизола в суточной моче (100–379 нмоль/сут) — иммунохемилюминесцентным методом на аппарате Vitros ECi; определение уровня калия (3,5–5,1 ммоль/л) проводилось на анализаторе Architect c 8000, Abbott, USA.

## Методы построения моделей

Концепция разработки и валидирования прогностических моделей представлена на рисунке 1.

**Figure:**
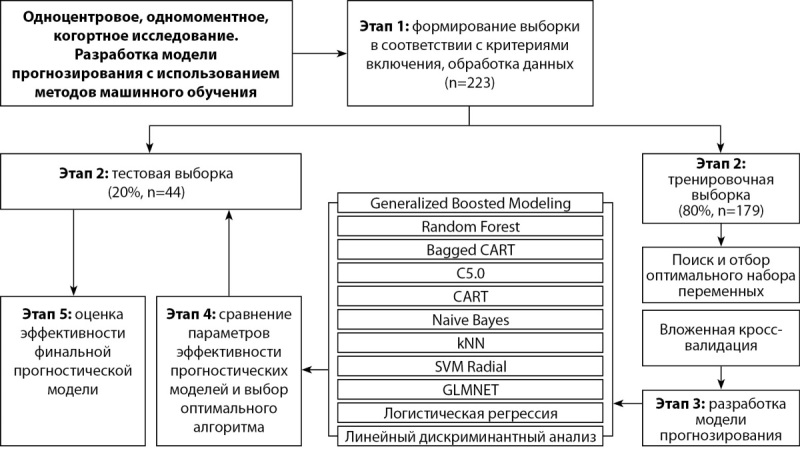
Рисунок 1. Концепция разработки прогностических моделей.

Предварительная обработка данных включала в себя удаление признаков с околонулевой дисперсией (near-zero variance), отсев коллинеарных предикторов. Кроме того, для тех алгоритмов, которые этого требуют, проводилась нормализация и центрирование предикторов. Во избежание утечки данных (data leakage) вся предварительная обработка и трансформация выполнялись после разделения набора данных.

Разделение на обучающую/тренировочную (training set) и тестовую (test set) выборки выполнялось по схеме 80/20 случайным стратифицированным отбором. С целью подтверждения соответствия распределения данных в обучающей и тестовой выборках проводился анализ с оценкой модифицированного многомерного расстояния Махаланобиса (Eklavya Jain, 2022) и тестированием нулевой гипотезы о соответствии распределений (методом Монте-Карло). Анализ показал небольшую дистанцию между обучающей и тестовой выборками — 12,7. Принимая нулевую гипотезу с уровнем значимости p-value 0,755, мы пришли к выводу, что данное разделение может быть использовано для измерения эффективности модели.

Имеющиеся данные подвергли математической обработке с задачей выбора оптимального набора показателей, которые с большей вероятностью были бы характерны для БИК или АКТГ-ЭС. Дополнительно проводился поиск параметров, рекомендуемых программой на основании математических расчетов, для оптимального управления процессом обучения модели. В качестве потенциальных параметров рассматривались исходные клинические признаки, представленные в таблице 1.

**Table:** Таблица 1. Общая характеристика и основные лабораторные показатели включенных в исследование пациентов с АКТГ-зависимым эндогенным гиперкортицизмом

Параметр	Пациенты с болезнью Иценко-Кушинга	Пациенты с АКТГ-эктопированным синдромом	р
Общее количество пациентов	175	48	–
Мужчины (%) : Женщины (%)	41 (23,4%) : 134 (76,6%)	19 (39,6%) : 29 (60,4%)	0,029*
Возраст на момент заболевания, лет	35 [ 27; 46] (11; 70)	36,5 [ 28; 54] (16; 76)	0,129
Возраст на момент включения в исследование, лет	39 [ 30; 50] (18; 72)	43 [ 31; 57] (18; 76)	0,213
Индекс массы тела, кг/м²	31,1 [ 26,6; 34,7] (18,3; 57,5)	29 [ 25,3; 33,6] (21; 48,7)	0,148
Лабораторные параметры на момент установки диагноза
Кортизол в крови в 23:00, нмоль/л	660,1 [ 518,2; 904] (93,2; 1960)	1128,5 [ 691,1; 1412,3] (460,2; 5854,8)	<0,001*
Кортизол в слюне в 23:00, нмоль/л	22,5 [ 14,7; 39,3] (4,2; 436)	60,1 [ 35,4; 117] (9,8; 711,5)	<0,001*
Кортизол в суточной моче, нмоль/сут	1198,4 [ 721; 2362] (284; 15196)	3460,4 [ 1597,6; 6994,2] (640,2; 12332,25)	<0,001*
АКТГ в 08:00, пг/мл	63,5 [ 45,5; 82,1] (13,4; 428,3)	127,2 [ 94,2; 188,3] (39,9; 536,7)	<0,001*
АКТГ в 23:00, пг/мл	51,5 [ 33,2; 75,3] (8,1; 208,7)	108,6 [ 77,9; 173,3] (38; 755,6)	<0,001*
Минимальный уровень калия в период заболевания, ммоль/л	4,2 [ 3,9; 4,4] (1,6; 5,1)	3,5 [ 2,8; 4,1] (1,6; 4,9)	<0,001*
Малая проба с 1 мг дексаметазона
Проба положительная	1	0	0,758
Проба отрицательная	173	48
Большая проба с 8 мг дексаметазона
Проба положительная	65	4	<0,001*
Проба отрицательная	14	26
Визуализация аденомы на МРТ головного мозга
С визуализацией аденомы	105	20	0,031*
Без визуализации аденомы	68	27
Максимальный размер аденомы на МРТ, мм	3,5 [ 0,0; 5,5] (0; 10)	0,0 [ 0,0; 4,5] (0; 8)	0,551
МРТ не выполнялось	2	1	–

Для разработки модели прогнозирования АКТГ-ЭС среди пациентов с АКТГ-зависимым эндогенным гиперкортицизмом проведено сравнение 11 алгоритмов машинного обучения: линейный дискриминантный анализ (Linear Discriminant Analysis), логистическая регрессия (Logistic Regression), эластичная сеть (GLMNET), метод опорных векторов (SVM Radial), метод k-ближайших соседей (k-nearest neighbors, kNN), наивный байесовский классификатор (Naive Bayes), бинарное дерево решений (CART), алгоритмы дерева решений C5.0, бутстрэп-агрегирование CART (Bagged CART), случайный лес (Random Forest), градиентный бустинг (Stochastic Gradient Boosting, GBM). Результатом прогнозирования является вероятность наличия у пациента АКТГ-ЭС, выраженная в процентах.

## Обучение моделей

Для идентификации наилучшего алгоритма прогнозирования АКТГ-ЭС среди пациентов с АКТГ-зависимым эндогенным гиперкортицизмом применялась вложенная кросс-валидация с 5 внешними и 3 внутренними циклами (3x5 Nested Cross-Validation) по схеме, предложенной Isci S. и соавт. [10]. Данный подход обеспечивает более точную оценку ошибки обобщения (Generalization error) при одновременной оптимизации гиперпараметров и показал свою применимость в предметной области.

Алгоритм Boruta (Kursa and Rudnicki, 2010) отвечал за отбор предикторов для обучения моделей. Данный алгоритм является оберточным методом отбора признаков и настроен на поиск минимального оптимального набора признаков вместо всех возможных релевантных признаков, что приводит к несмещенному и устойчивому отбору важных предикторов.

Обучение и оценка производительности алгоритмов машинного обучения проводились на тренировочном наборе данных. После выбора наилучшего алгоритма осуществлялась его финальная оценка на тестовом наборе.

## Оценка производительности моделей

Результаты эффективности полученных моделей сравнивались между собой путем анализа стандартных метрик, полученных из матрицы ошибок классификации (confusion matrix): 1) аккуратность (Accuracy); 2) площадь под кривой операционных характеристик — ROC (Receiver Operating Characteristic curve) AUC (Area Under Curve); 3) чувствительность (Sensitivity); 4) специфичность (Specificity); 5) точность (Precision); 6) полнота (Recall); 7) F₁-мера.

Основными метриками для выбора финальной модели были ROC-AUC, чувствительность и специфичность.

## Статистический анализ

При анализе исходных данных количественные показатели представлены с помощью медианы (Me) с указанием интерквартильного диапазона [Q25–Q75], максимальных и минимальных значений, качественные переменные представлены в виде абсолютных и относительных частот. Соотношения качественных признаков представлены в виде долей (%). Сравнение двух независимых групп для количественных данных выполнялось с помощью критерия Стьюдента для признаков, соответствующих закону нормального распределения, и критерия Манна-Уитни для признаков, не соответствующих закону нормального распределения. Качественные переменные сравнивались между собой с помощью критерия хи-квадрат (χ2) и точного двустороннего критерия Фишера.

Обработка данных осуществлялась при помощи пакета статистических программ IBM SPSS Statistics 23 (SPSS. Inc, Chicago, IL, USA).

Построение и разработка прогностических моделей, оценка их производительности выполнялись с использованием пакета программ R версии 4.2.3.

## Этическая экспертиза

Протокол исследования одобрен локальным этическим комитетом ГНЦ РФ ФГБУ «НМИЦ эндокринологии» Минздрава России, выписка из протокола №2 от 20.02.2013 г., №12 от 29.06.2022 г. Все пациенты, включенные в исследование, подписали информированное согласие на участие в исследовании.

## РЕЗУЛЬТАТЫ

## Объекты (участники) исследования

В исследование включено 223 пациента (163 женщины, 60 мужчин) с АКТГ-зависимым эндогенным гиперкортицизмом, из них 175 пациентов с БИК, 48 — с АКТГ-ЭС. Общая характеристика пациентов, основные лабораторные параметры и результаты сравнительного анализа представлены в таблице 1. Больные распределены на две группы по окончательному клиническому диагнозу.

На первом этапе исследования анализировали возможные различия клинико-демографических и лабораторно-инструментальных показателей в группах сравнения (табл. 1). Пациенты обеих групп были преимущественно женского пола, не различались по возрасту на момент заболевания, индексу массы тела и результатам малой дексаметазоновой пробы (МДП). Больные с АКТГ-ЭС имели более высокие уровни всех основных гормональных показателей (р<0,001) и более низкие значения калия (р<0,001) в активной стадии заболевания. Отрицательные результаты БДП и отсутствие визуализации аденомы гипофиза по данным МРТ головного мозга статистически значимо чаще наблюдались в группе пациентов с АКТГ-ЭС в сравнении с пациентами с БИК (табл. 1). Из 223 пациентов трем больным не проведена визуализация гипофиза методом МРТ ввиду наличия кардиостимулятора, клаустрофобии, морбидного ожирения (ИМТ=57,5 кг/м²).

## Модель машинного обучения для прогнозирования вероятности АКТГ-ЭС у пациентов с АКТГ-зависимым эндогенным гиперкортицизмом

Исходно общая группа больных (n=223) случайным образом разделена на две выборки в соотношении 80:20% — для обучения (n=179) и для тестирования моделей (n=44) (рис. 1).

В результате предварительной обработки данных и отбора наиболее информативных признаков с помощью метода случайного леса и алгоритма Boruta финальными переменными для классификации и прогнозирования АКТГ-ЭС отобраны: уровень АКТГ в 08:00, уровень калия, показатели кортизола в суточной моче, кортизола в крови в 23:00, кортизола в слюне в 23:00, наибольший размер аденомы гипофиза на МРТ головного мозга. По окончании процедуры отбора каждый признак получил значение, которое выражает степень его информативности (рис. 2). Чем выше значение, тем ценнее этот признак для прогнозирования и классификации. В результате получен перечень признаков, которые можно ранжировать в соответствии с их значимостью для решения задачи классификации и прогнозирования (рис. 2).

**Figure:**
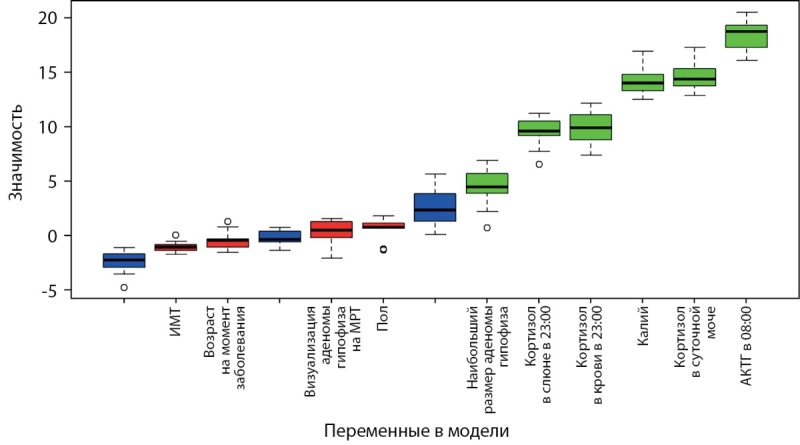
Рисунок 2. Ранжирование предикторов с использованием алгоритма Boruta.

Переменные, оказавшиеся менее релевантными, удалены из анализа: возраст на момент заболевания, пол, ИМТ, результат БДП, визуализация аденомы гипофиза на МРТ головного мозга (рис. 2).

В таблице 2 представлены параметры эффективности прогностических моделей, полученные после применения алгоритмов машинного обучения к тренировочной выборке (n=179). Сравнительный анализ индикаторов прогностической точности позволил выявить определенные различия моделей. Так, качество прогноза по метрикам AUC, чувствительности и специфичности для модели, реализованной на основе бинарного дерева решений (CART), составило 0,694; 87,3 и 48,7% соответственно, что свидетельствует о недостаточной точности модели при апробации на анализируемой когорте пациентов и необходимости ее совершенствования. В свою очередь модель GLMNET обеспечила на тренировочной выборке хорошую чувствительность (96,4%) и AUC (0,840) на фоне самой низкой специфичности (28,2%). Наиболее высокие значения ROC-AUC были получены для модели Naive Bayes и модели градиентного бустинга (AUC — 0,856 и 0,867 соответственно), что соответствует очень хорошей прогностической точности данных моделей.

**Table:** Таблица 2. Сравнение эффективности моделей в прогнозировании АКТГ-ЭС среди пациентов с АКТГ-зависимым гиперкортицизмом

Алгоритм	Площадь под ROC-кривой (AUC)	Чувствительность, %	Специфичность, %
Linear Discriminant Analysis	0,820	95,7	35,9
Logistic Regression	0,807	93,6	41,0
GLMNET	0,840	96,4	28,2
SVM Radial	0,824	91,4	51,3
kNN	0,783	95,7	28,2
Naive Bayes	0,856	95,0	48,7
CART	0,694	87,3	48,7
C5.0	0,818	84,9	61,5
Bagged CART	0,808	85,7	56,4
Random Forest	0,841	89,3	51,3
Generalized Boosted Modeling	0,867	90,0	56,4

Наилучшую предсказательную способность из всех обученных моделей машинного обучения по всем трем итоговым метрикам (ROC-AUC, чувствительность, специфичность) продемонстрировала модель градиентного бустинга (Generalized Boosted Modeling, GBM).

Финальная модель была обучена со следующими гиперпараметрами (табл. 3).

**Table:** Таблица 3. Финальные гиперпараметры модели градиентного бустинга (Generalized Boosted Modeling, GBM) Примечание: n.trees — число деревьев; interaction.depth — число внутренних узлов; shrinkage — параметр сжатия; n.minobsinnode — минимальное количество выборок в терминальных узлах дерева.

n.trees	interaction.depth	shrinkage	n.minobsinnode
50	3	0,1	10

Результаты проверки точности классификации, выполненной моделью градиентного бустинга (Generalized Boosted Modeling, GBM), на тестовой выборке (n=44) представлены на рисунках 3, 4, в таблице 4. Точность разработанной нами модели дифференциальной диагностики составляет: диагностика АКТГ-ЭС — с вероятностью верного заключения (прогностической ценностью положительного результата) 87,5%; исключение АКТГ-ЭС — с вероятностью верного заключения (прогностическая ценность отрицательного результата) 94,4%. Данный способ дифференциальной диагностики с использованием алгоритма машинного обучения позволяет с чувствительностью 77,8% и специфичностью 97,1% установить диагноз АКТГ-ЭС.

**Figure:**
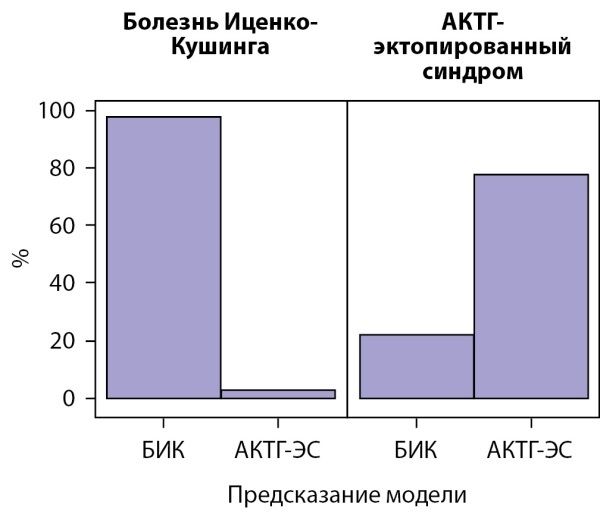
Рисунок 3. Точность предсказаний модели градиентного бустинга (Generalized Boosted Modeling, GBM) на тестовой выборке.

**Figure:**
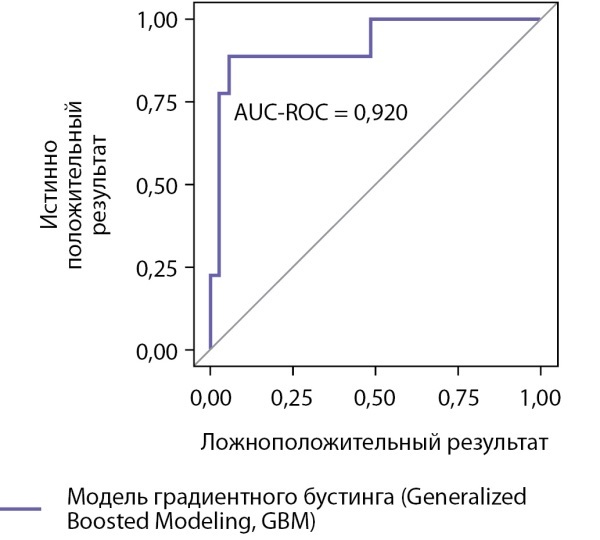
Рисунок 4. ROC-кривая для модели градиентного бустинга (Generalized Boosted Modeling, GBM), полученная на тестовой выборке.

**Table:** Таблица 4. Метрики качества прогностической модели градиентного бустинга Generalized Boosted Modeling (GBM), полученные после применения к тестовой выборке

Модель	Аккуратность (Accuracy)	F1-мера	Чувствительность	Специфичность	Точность (Precision)	Полнота (Recall)	ROC-AUC
GBM	93,2%	82,3%	77,8%	97,1%	87,5%	77,8%	0,920

Значимость клинических признаков, определяющих точность прогнозирования для модели градиентного бустинга (Generalized Boosted Modeling, GBM), показана на рисунке 5. Выявлено, что доминирующее воздействие на результирующую переменную оказывают 6 факторов, при этом к наиболее значимым параметрам относятся уровень АКТГ в ранние утренние часы, минимальное значение калия в активной стадии заболевания, уровень кортизола в суточной моче и кортизола в крови в 23:00. Меньшее влияние оказывали показатели свободного кортизола в слюне, собранной в 23:00, а вклад наибольшего диаметра аденомы гипофиза по результатам МРТ головного мозга был минимальным (рис. 5).

**Figure:**
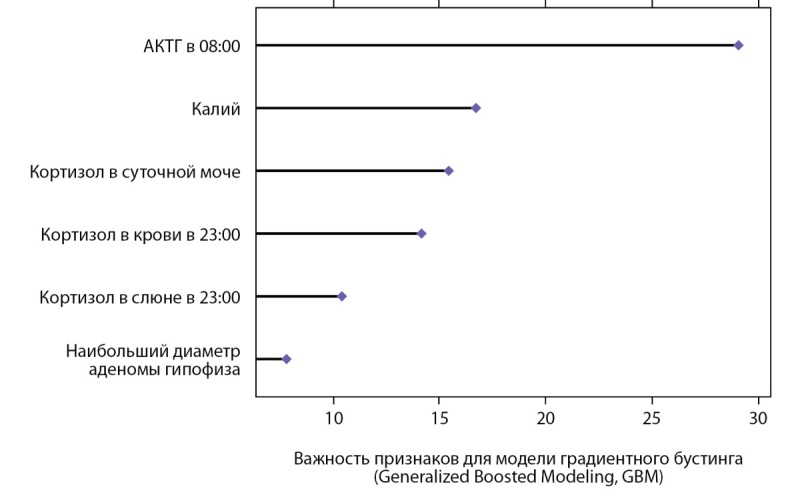
Рисунок 5. Прогностическая важность признаков, обеспечивающих принятие решения для модели градиентного бустинга (Generalized Boosted Modeling, GBM).

## Примеры использования разработанной модели машинного обучения в дифференциальной диагностике АКТГ-зависимого эндогенного гиперкортицизма

Клинический пример 1

Пациент С., 38 лет, поступил в отделение нейроэндокринологии и остеопатий ФГБУ «НМИЦ эндокринологии» Минздрава России. Результаты обследования пациента С. в стационаре представлены в таблице 5. По данным МРТ головного мозга с внутривенным контрастированием, выявлена диффузная неоднородность структуры аденогипофиза, убедительных данных за микроаденому гипофиза не получено.

**Table:** Таблица 5. Результаты обследований пациента С.

Параметр	Значение
АКТГ в крови в 08:00, пг/мл	82,4
Кортизол в крови в 23:00, нмоль/л	653,2
Кортизол в слюне в 23:00, нмоль/л	52,7
Кортизол в суточной моче, нмоль/сут	2538,4
Калий, ммоль/л	3,7
Наибольший диаметр аденомы гипофиза по данным МРТ головного мозга, мм	0

Согласно расчету разработанной нами прогностической модели, у данного пациента вероятность АКТГ-ЭС составляет 11%, то есть в данном случае модель позволяет сделать вывод в пользу наличия у пациента С. БИК.

Для подтверждения полученного вывода с помощью разработанной модели машинного обучения мы сравнили результаты гистологического и иммуногистохимического исследований удаленной опухолевой ткани гипофиза пациента С. Гистологическое заключение: аденома гипофиза из базофильных клеток. Иммуногистохимическое заключение: кортикотропинома (положительная экспрессия АКТГ). Таким образом, заключения, сделанные на основании разработанного нами неинвазивного метода дифференциальной диагностики с использованием алгоритма машинного обучения и результатов патоморфологического исследования, совпали.

Клинический пример 2

Пациентка О., 32 года, поступила в отделение нейроэндокринологии и остеопатий ФГБУ «НМИЦ эндокринологии» Минздрава России. Результаты обследования пациентки О. в стационаре представлены в таблице 6. По данным МРТ головного мозга с внутривенным контрастированием — микроаденома гипофиза размерами 5,5х4 мм.

**Table:** Таблица 6. Результаты обследований пациентки О.

Параметр	Значение
АКТГ в крови в 08:00, пг/мл	133,9
Кортизол в крови в 23:00, нмоль/л	809
Кортизол в слюне в 23:00, нмоль/л	47,08
Кортизол в суточной моче, нмоль/сут	5542
Калий, ммоль/л	4,06
Наибольший диаметр аденомы гипофиза по данным МРТ головного мозга, мм	5,5

Согласно расчету модели, у данной пациентки вероятность АКТГ-ЭС составила 62%, что привело к необходимости проведения дополнительных инструментальных методов диагностики с целью локализации источника эктопической гиперпродукции АКТГ. По результатам МСКТ органов грудной клетки выявлено образование в S4 правого легкого размерами 7,5х5 мм. При проведении соматостатин-рецепторной сцинтиграфии, совмещенной с ОФЭКТ/КТ, с 99mTc-Тектротидом, — аналогичное образование в S4 правого легкого с признаками повышенной фиксации 99mTc-Тектротида. Для подтверждения полученного вывода мы сравнили результаты гистологического и иммуногистохимического исследований удаленной опухолевой ткани легкого. Гистологическое заключение: типичный карциноид легкого. Иммуногистохимическое заключение: в клетках опухоли очаговая экспрессия АКТГ. Таким образом, разработанная нами модель машинного обучения верно классифицировала форму АКТГ-зависимого гиперкортицизма у данной пациентки, что подтверждено результатами патоморфологического исследования удаленной опухолевой ткани легкого.

## ОБСУЖДЕНИЕ

В настоящем исследовании впервые в РФ разработана модель с использованием методов машинного обучения и предложен способ неинвазивной дифференциальной диагностики АКТГ-зависимых форм эндогенного гиперкортицизма (БИК и АКТГ-ЭС) с предсказанием вероятности наличия у пациента АКТГ-ЭС, характеризующийся высокой точностью (93,2%), чувствительностью (77,8%) и специфичностью (97,1%). Использование разработанного нами алгоритма способно существенно снизить потребность в проведении инвазивной дифференциальной диагностики с применением метода двустороннего селективного забора крови из нижних каменистых синусов.

Прогностические исследования в клинической медицине относятся к одним из перспективных и быстро развивающихся направлений. Данные зарубежной литературы свидетельствуют о возрастающем интересе к разработке прогностических моделей неинвазивной верификации этиологии эндогенного гиперкортицизма на основе современных технологий машинного обучения. В настоящее время активно проводятся исследования, направленные на совершенствование и повышение предсказательной ценности моделей. Например, модели машинного обучения позволяют эффективно прогнозировать как ранние послеоперационные исходы у больных с аденомой гипофиза, включая пациентов с БИК [11], так и долгосрочную ремиссию после удаления кортикотропиномы гипофиза [12]. Однако наименьшее количество исследований в этой области посвящено дифференциальной диагностике различных форм гиперкортицизма.

Isci S. и соавт. [10] впервые предложили способ дифференциальной диагностики различных форм эндогенного гиперкортицизма (БИК, синдром Иценко-Кушинга (СИК), субклинический гиперкортицизм) с использованием методов машинного обучения, основанный на данных 241 пациента и включивший 11 переменных-предикторов. Для создания прогностических моделей ученые применили различные методы машинного обучения, по результатам которых алгоритм случайного леса (Random Forest, RF) значительно превосходил прогностические способности других алгоритмов машинного обучения. Применение модели позволило классифицировать синдром гиперкортицизма со средней точностью 92%. Алгоритм бинарной классификации на основе RF «один против всех» (one vs all) продемонстрировал чувствительность 97,6%, точность 91,1% и специфичность 87,1% в подтверждении либо исключении эндогенного гиперкортицизма в тестовой выборке. Мультиклассовая классификация с использованием RF имела среднюю чувствительность по классам 91,8%, среднюю специфичность 97,1% и среднюю точность 92,1% в классификации различных вариантов эндогенного гиперкортицизма в тестовом наборе данных. Однако разработанный зарубежными исследователями алгоритм не позволяет проводить дифференциальную диагностику БИК и АКТГ-ЭС ввиду отсутствия в выбранной когорте больных с АКТГ-ЭС.

Впервые исследования возможности методов машинного обучения в области дифференциальной диагностики АКТГ-зависимых форм эндогенного гиперкортицизма (БИК и АКТГ-ЭС) проведены китайскими учеными Lyu X. и соавт. [13][14]. Данные 311 пациентов, включая 47 больных с АКТГ-ЭС, были использованы для моделирования. На основании многомерного логистического регрессионного анализа из одиннадцати переменных женский пол (отношение шансов (ОШ) — 3,030), гипокалиемия (ОШ — 0,209), уровень АКТГ (ОШ — 0,988), визуализация аденомы гипофиза на МРТ (ОШ — 8,671)и положительный результат БДП (ОШ — 2,768) идентифицированы как предикторы, ассоциированные с БИК, у пациентов с АКТГ-зависимым эндогенным гиперкортицизмом [13]. Из 8 созданных моделей GBM давала наибольшие значения площади под ROC-кривой (AUC 0,980±0,02), однако модель RF оказалась наилучшей для прогнозирования БИК среди всех моделей машинного обучения. Чувствительность алгоритма RF составила 98,9%, специфичность — 87,9%, AUC — 0,976. Применение прогностической модели к обучающей выборке продемонстрировало высокую чувствительность (98,4%) и специфичность (100%) разработанного алгоритма, однако в тестовом наборе данных показатели чувствительности и специфичности составили 95% и 71,4% соответственно. По результатам исследования, тремя наиболее значимыми переменными оказались уровень калия в сыворотке крови, размер аденомы гипофиза на МРТ и значения АКТГ. Модель, основанная на алгоритме RF, имела наибольшую площадь под ROC-кривой (AUC 0,984 (95% ДИ 0,950–0,993)) в сравнении с AUC для МДП и БДП (p<0,001), однако не выявлено статистически значимых различий при сравнении площадей выбранной модели и метода «золотого стандарта» — двустороннего селективного забора крови из нижних каменистых синусов с использованием стимуляционного агента [14].

В отличие от вышеупомянутого исследования, в нашей работе к универсальным предикторам, определяющим высокую точность прогнозирования, помимо уровня АКТГ в ранние утренние часы и калия, относились такие показатели, как концентрация свободного кортизола в суточной моче, кортизола в крови в 23:00, кортизола в образце слюны, собранной в 23:00, и наибольший диаметр аденомы гипофиза по результатам МРТ головного мозга. Считается, что для АКТГ-ЭС характерно более тяжелое течение с быстрым нарастанием выраженности клинической симптоматики и более высокие уровни всех основных гормональных показателей [15]. Снижение уровня калия в крови встречается при гиперкортицизме любой этиологии и не является специфическим проявлением АКТГ-ЭС несмотря на то, что частота развития гипокалиемии при синдроме эктопической продукции АКТГ может достигать 82,6% против 21% случаев при БИК (р=0,001), согласно результатам сравнительного исследования Attri B. и соавт. [15]. По нашим данным, результаты дооперационного лабораторного обследования статистически значимо различались между пациентами с БИК и АКТГ-ЭС (p<0,001), что согласуется с вышеупомянутым исследованием.

В настоящей работе прогнозирование вероятности АКТГ-ЭС базировалось на результатах анализа простых лабораторных и инструментальных параметров, доступных в обычной клинической практике. Высокое качество переменных-предикторов в нашем исследовании обеспечивалось многоступенчатой процедурой отбора, включавшей оценку их информативности с помощью автоматических методов, в частности алгоритма Boruta, а также подбор клинических признаков, определяющих точность прогнозирования. Использование данного алгоритма позволило ранжировать отдельные предикторы по степени влияния на прогностический потенциал и определить переменные, необходимые для построения высокопроизводительной модели.

Результаты нашего исследования согласуются с выводами зарубежных ученых, согласно которым более сложные методы машинного обучения превосходят возможности традиционных статистических моделей, включая модели на основе логистической регрессии. Однако, в отличие от ранее опубликованных исследований, в нашей работе наилучшие метрики качества в прогнозировании вероятности АКТГ-ЭС наблюдались при применении метода машинного обучения, реализованного при помощи градиентного бустинга (Generalized Boosted Modeling, GBM), в связи с чем данная модель представляется предпочтительной для использования в клинической практике.

## Клиническая значимость результатов

Разработанная в настоящем исследовании модель дифференциальной диагностики и предсказания вероятности АКТГ-ЭС может быть реализована программным путем (в виде калькулятора) и представляет собой инструмент для поддержки принятия решения. Калькулятор может включать поля с возможностью введения значений показателей (результаты лабораторных исследований — АКТГ в ранние утренние часы, кортизол в крови в 23:00, свободный кортизол в слюне в 23:00, кортизол в суточной моче, калий; результат МРТ головного мозга — наибольший диаметр аденомы гипофиза). На основании полученного результата врач сможет персонифицировать алгоритм ведения пациента, выбрать оптимальный объем лечебных и, в случае положительного прогноза модели, дополнительных диагностических мероприятий, что в конечном итоге позволит повысить эффективность лечения, снизит сроки временной нетрудоспособности, проведение неоправданных оперативных вмешательств и процент инвалидизации пациентов.

## Ограничения исследования

К ограничениям данного исследования следует отнести сравнительно небольшую выборку, связанную в первую очередь с орфанностью основного заболевания (БИК и АКТГ-ЭС). Одним из ограничений также является одноцентровой дизайн исследования, что может способствовать снижению эффективности предсказательной ценности модели при внешней валидации.

## Направления дальнейших исследований

Перспективы дальнейших исследований связаны с расширением обучающей выборки за счет привлечения данных из других медицинских учреждений РФ, что будет способствовать повышению точности модели.

## ЗАКЛЮЧЕНИЕ

Прогностическая модель, основанная на методах машинного обучения, позволяет дифференцировать пациентов с АКТГ-ЭС и БИК по базовым клиническим результатам и может быть использована в качестве первичного скрининга больных с АКТГ-зависимым эндогенным гиперкортицизмом. Полученные результаты оправдывают использование методов машинного обучения в области медицинского прогнозирования, являются шагом в сторону разработки персонализированного подхода к выбору метода лечения и могут служить основой для реализации систем поддержки принятия врачебных решений у пациентов с АКТГ-зависимым гиперкортицизмом.

## ДОПОЛНИТЕЛЬНАЯ ИНФОРМАЦИЯ

Источники финансирования. Исследование проведено при поддержке Российского научного фонда (грант РНФ 19-15-00398-П).

Конфликт интересов. Авторы декларируют отсутствие явных и потенциальных конфликтов интересов, связанных с содержанием настоящей статьи.

Участие авторов. Голоунина О.О. — сбор и обработка полученных данных, формирование электронной базы данных, статистическая обработка, анализ и интерпретация полученных результатов, написание основного текста; Белая Ж.Е. — концепция и дизайн исследования, научное руководство проводимого исследования, ведение пациентов, редактирование текста; Воронов К.А., Солодовников А.Г. — статистическая обработка данных, математическое моделирование, разработка алгоритма машинного обучения, написание и редактирование текста; Рожинская Л.Я. — ведение пациентов, редактирование текста; Мельниченко Г.А., Мокрышева Н.Г., Дедов И.И. — редактирование текста, одобрение финальной версии рукописи. Все авторы одобрили финальную версию статьи перед публикацией, выразили согласие нести ответственность за все аспекты работы, подразумевающую надлежащее изучение и решение вопросов, связанных с точностью или добросовестностью любой части работы.
